# Machine learning-based identification of the novel circRNAs circERBB2 and circCHST12 as potential biomarkers of intracerebral hemorrhage

**DOI:** 10.3389/fnins.2022.1002590

**Published:** 2022-11-29

**Authors:** Congxia Bai, Xiaoyan Hao, Lei Zhou, Yingying Sun, Li Song, Fengjuan Wang, Liu Yang, Jiayun Liu, Jingzhou Chen

**Affiliations:** ^1^Department of Clinical Laboratory Medicine, Xijing Hospital, Fourth Military Medical University, Xi’an, China; ^2^State Key Laboratory of Cardiovascular Disease, Fuwai Hospital, National Center for Cardiovascular Diseases, Chinese Academy of Medical Sciences and Peking Union Medical College, Beijing, China; ^3^National Health Commission Key Laboratory of Cardiovascular Regenerative Medicine, Fuwai Central-China Hospital, Central-China Branch of National Center for Cardiovascular Diseases, Zhengzhou, China

**Keywords:** intracerebral hemorrhage, RNA sequencing, circular RNA, biomarkers, machine learning algorithms

## Abstract

**Background:**

The roles and potential diagnostic value of circRNAs in intracerebral hemorrhage (ICH) remain elusive.

**Methods:**

This study aims to investigate the expression profiles of circRNAs by RNA sequencing and RT–PCR in a discovery cohort and an independent validation cohort. Bioinformatics analysis was performed to identify the potential functions of circRNA host genes. Machine learning classification models were used to assess circRNAs as potential biomarkers of ICH.

**Results:**

A total of 125 and 284 differentially expressed circRNAs (fold change > 1.5 and FDR < 0.05) were found between ICH patients and healthy controls in the discovery and validation cohorts, respectively. Nine circRNAs were consistently altered in ICH patients compared to healthy controls. The combination of the novel circERBB2 and circCHST12 in ICH patients and healthy controls showed an area under the curve of 0.917 (95% CI: 0.869–0.965), with a sensitivity of 87.5% and a specificity of 82%. In combination with ICH risk factors, circRNAs improved the performance in discriminating ICH patients from healthy controls. Together with hsa_circ_0005505, two novel circRNAs for differentiating between patients with ICH and healthy controls showed an AUC of 0.946 (95% CI: 0.910–0.982), with a sensitivity of 89.1% and a specificity of 86%.

**Conclusion:**

We provided a transcriptome-wide overview of aberrantly expressed circRNAs in ICH patients and identified hsa_circ_0005505 and novel circERBB2 and circCHST12 as potential biomarkers for diagnosing ICH.

## Introduction

Stroke causes high levels of mortality and disability globally. Intracerebral hemorrhage (ICH) is a deadly stroke subtype with an estimated annual incidence of 16 per 100,000 persons worldwide (Wilkinson et al., 2018). ICH accounts for approximately 23.8% of stroke cases in China, compared with Western countries, where it accounts for 10–15% of stroke cases, causing a median fatality ratio of 40.4% per month (Qureshi et al., 2009; Benjamin et al., 2017). The diagnosis of stroke is often made with computed tomography (CT) or magnetic resonance imaging (MRI), and although most patients are hospitalized with typical neurological symptoms, it is difficult to distinguish ICH from ischemic stroke (IS) in the super acute period (Hankey, 2017). Thus, identifying potential biomarkers for the early prediction and diagnosis of ICH is important.

Non-coding RNAs (ncRNAs) have been extensively studied in the pathophysiology of cerebrovascular diseases (Weng et al., 2022). Changes in RNA levels during stroke have the potential to aid stroke diagnosis and provide insight into stroke diagnosis and management (Montaner et al., 2020). Emerging evidence has revealed that ncRNA expression profiles are altered in the peripheral blood of patients with ICH (Kim et al., 2019; Li et al., 2019; Cheng et al., 2020). CircRNAs are a novel class of ncRNAs that are produced in eukaryotic cells during posttranscriptional processes; these covalently closed RNAs lack a free 3′ or 5′ end and are resistant to exonuclease digestion (Kristensen et al., 2019). Thus, circRNAs are promising diagnostic and prognostic biomarkers for many human diseases because of their stability, specificity and abundance in human blood (Jeck and Sharpless, 2014; Zhang et al., 2018). Growing evidence has demonstrated that circRNAs are implicated in a variety of pathological conditions, including coronary artery disease (Cardona-Monzonis et al., 2020), acute ischemic stroke (Liu Y. et al., 2022) and cancers (Kristensen et al., 2022). Moreover, the expression of circRNAs was found to be significantly altered in IS (Tiedt et al., 2017; Dong et al., 2020; Li et al., 2020; Lu et al., 2020; Ostolaza et al., 2020; Zuo et al., 2020), and these studies implied that aberrantly expressed circRNAs may be novel biomarkers for IS diagnosis and prognosis. Our previous study revealed that circRNA profiles were significantly altered in hypertensive ICH patients compared to hypertensive subjects without ICH and found that hsa_circ_0001240, hsa_circ_0001947 and hsa_circ_0001386 were potential biomarkers for predicting and diagnosing hypertensive ICH (Bai et al., 2021). In addition, circRNA expression is significantly altered in rat brain tissue after ICH (Dou et al., 2020; Zhong et al., 2020), indicating that circRNAs are novel clinical biomarkers for ICH. However, comprehensive circRNA expression profiles and their potential diagnostic value in the peripheral blood of ICH patients remain elusive.

Artificial intelligence techniques such as machine learning tools have been increasingly used in precision diagnosis (Chang et al., 2021). Machine learning algorithms are artificial intelligence techniques used to select the best model from a set of alternatives to fit a set of observations (Li, 2018). Machine learning has remained a fundamental and indispensable tool due to its efficacy and efficiency in both feature extraction of relevant biomarkers and the classification of samples as validation of the discovered biomarkers (Ledesma et al., 2021).

In this study, we investigated the expression profile of circRNAs in peripheral blood cells from patients with ICH, patients with IS and healthy controls by RNA sequencing in the discovery and validation cohorts. The significantly altered circRNA host genes were examined with Gene Ontology (GO) and Kyoto Encyclopedia of Genes and Genomes (KEGG) pathway analyses to characterize the potential functions. We further validated the altered circRNAs by quantitative reverse transcription-PCR (RT–PCR) analysis of all samples. Logistic regression models were performed to identify whether circRNAs were independent factors for ICH. Additionally, we performed Spearman’s correlation analysis to investigate the correlation between ICH risk factors and candidate circRNAs. Furthermore, machine learning classification algorithms and ROC curves were used to assess circRNAs as potential biomarkers of ICH.

## Materials and methods

### Study design and sample collection

We recruited 64 patients with ICH, 59 patients with IS and 50 sex- and age-matched healthy controls between 2014 and 2019 from two individual cohorts for RNA sequencing. In the discovery cohort, 44 patients with ICH, 43 patients with IS and 31 healthy controls were enrolled from Cangzhou Central Hospital between 2014 and 2017. In the validation cohort, 20 patients with ICH were enrolled from the Affiliated Hospital of Hebei University, 16 patients with IS were enrolled from General Hospital of Ningxia Medical University, and 19 healthy control subjects were enrolled from the Tsinghua University Hospital between 2017 and 2019. Patients with ICH were diagnosed by professional neurologists based on their histories and examinations, and ICH was confirmed by CT or MRI. Healthy controls without a history of stroke or cardiovascular events were selected. The demographic and clinical characteristics of the study population were obtained through a face-to-face survey and by checking hospital records or medical examination records. The exclusion criteria included autoimmune diseases, cardiac disease, liver diseases, renal diseases, cancer or a history of stroke and cerebral infarction with hemorrhagic transformation. This study was reviewed and approved by the Human Ethics Committee, Fuwai Hospital (Approval No. 2016-732), and conducted in accordance with the principles of Good Clinical Practice and the Declaration of Helsinki. Written informed consent was obtained from all participants or their legal proxies.

### RNA isolation and sequencing

RNA was isolated from human peripheral blood and used to perform RNA sequencing by Annoroad Gene Technology Company Ltd. (Beijing, China), as previously described (Bai et al., 2021). Total RNA from all samples was isolated with an RNeasy Mini kit (QIAGEN). An Agilent 2100 RNA Nano 6000 Assay Kit (Agilent Technologies, CA, USA) was used to measure RNA integrity. The libraries were constructed using an RNA integrity number ≥7.5 and a 28S:18S rRNA ratio ≥ 1.8. Ribo-Zero™ Gold Kits (Illumina, San Diego, CA, USA) were utilized to eliminate all ribosomal RNAs from total RNA. RNase R (Epicenter, Madison, WI, USA) digestion was used to eliminate linear RNAs. The purified circRNAs were subjected to the NEB Next Ultra Directional RNA Library Prep Kit for Illumina (NEB, Ipswich, USA) according to the manufacturer’s instructions. The obtained libraries were subjected to paired-end sequencing with 150 bp reads performed on the Illumina PE150 platform. The sequence depth was approximately 15G. The raw sequencing data were analyzed using Q30 statistics from FastQC, and clean reads were obtained by removing adaptor-polluted and low-quality reads. The RNA-seq data have been deposited into the Genome Sequence Archive (Chen T. et al., 2021) in the National Genomics Data Center (CNCB-NGDC Members and Partners, 2022), China National Center for Bioinformation/Beijing Institute of Genomics, Chinese Academy of Sciences (GSA-Human: HRA001807), which are publicly accessible at https://ngdc.cncb.ac.cn/gsa-human.

### Differential expression analysis

The differential expression circRNA analysis was performed as previously described (Bai et al., 2021). Briefly, CIRI2 (Gao et al., 2018) was used to detect paired chiastic clipping signals according to the mapping of reads. The reads were mapped to the reference genome^1^ using the BWA-MEM method. Back-spliced junction reads were integrated and measured by spliced reads per billion mapping to quantify circRNA. Differential expression analysis was performed using the DESeq2 R package (Wang et al., 2010) and edgeR (Robinson et al., 2010). Fold differences of each circRNA were calculated to identify differentially expressed circRNAs between ICH patients and healthy controls (or IS patients) by Student’s *t*-test. A P value was assigned to each circRNA and adjusted by multiple testing using the Benjamini–Hochberg method for controlling the false discovery rate (FDR). The differentially expressed circRNAs were defined as those with a fold change ≥ 1.5 and FDR < 0.05.

### Bioinformatics analysis

Volcano plots and hierarchical clustering using heatmaps were generated based on the normalized values of differentially expressed genes using the R package. Venn diagrams were used to present the consistently differentially expressed genes in the discovery and validation cohorts. GO enrichment and KEGG analyses were performed to determine the biological functions and pathways of differentially expressed circRNA host genes. P values were calculated using Fisher’s exact test with the hypergeometric algorithm.

### Quantitative real-time polymerase chain reaction validation

To validate the expression levels of differentially expressed circRNAs identified by RNA-seq, the candidate circRNAs were selected for further validation of expression levels by quantitative RT–PCR. Total RNA was incubated with RNase R or RNase-free water as a control at 37°C for 30 min to purify the circRNAs. After incubation, cDNA synthesis was completed using 1 μg of total RNA and a Transcriptor First Stand cDNA Synthesis Kit (Takara, Dalian, China), and Taq premix (Takara, Dalian, China) was added to start PCR according to the manufacturer’s protocol. The products were used for Sanger sequencing. Quantitative RT–PCR was performed using SYBR Master Mix (Yeasen, Shanghai, China) on the ViiA 7 Real-time PCR System (Applied Biosystems) according to the manufacturer’s instructions. The circRNA primers were designed to overlap the back-spliced junction using the NCBI Primer-BLAST website.^2^ The primers used in this study are listed in Supplementary Table 7. The relative expression of the corresponding genes was quantified and normalized to that of GAPDH.

### Performance evaluation of candidate biomarkers with classification algorithms

To evaluate the applicable biomarkers for ICH, we used mutual information (MI) (Blokh and Stambler, 2017) and random forest (RF) algorithms (Ambale-Venkatesh et al., 2017; Kawakami et al., 2019) to screen circRNA biomarker signatures according to the expression levels in all samples. To assess the diagnostic values of the specific circRNAs, we used six machine learning classification algorithms (Chang et al., 2021; Chen Y. et al., 2021; Liu D. et al., 2022), support vector machine (SVM), RF, K-nearest neighbor (KNN), logistic regression (LR), decision tree (DT) and Gaussian naive Bayes (GNB), to discriminate ICH patients from healthy controls or IS patients according to the expression levels of circRNAs by Python packages. To ensure the stability and accuracy of the classifiers, we used 10-fold cross-validation; 90% of the data were used for the training set, and 10% were used for the test set. We calculated five measurements, including sensitivity, specificity, accuracy, positive predictive value (PPV), and negative predictive value (NPV) (Shu et al., 2020). The ROC curve was illustrated based on sensitivity and 1-specificity scores. For each area under the curve (AUC) value, the 95% CI was computed with 1000 stratified bootstrap replicates.

### Statistical analysis

Statistical analysis was performed using SPSS 21.0 (IBM Corp., NY, USA). The sample distribution was determined using the Kolmogorov–Smirnov normality test. For parametric data, the two-tailed unpaired Student’s *t*-test was used to determine differences between two groups. The data are represented as the means ± standard deviations or medians (interquartile range). Statistical comparisons for percentages were performed using chi-square statistical analysis. In the RNA sequencing analysis, differentially expressed RNAs were selected if there were significant differences (fold change > 1.5 and FDR < 0.05) between the ICH patients and healthy controls (or IS patients) using Student’s *t*-test. Logistic regression models were used to evaluate whether circRNAs were independent predictive factors for ICH. Spearman’s correlation analysis was performed to investigate the correlation between ICH risk factors and circRNAs. The net reclassification index (NRI) and integrated discrimination improvement (IDI) were calculated to evaluate the effect of the candidate biomarkers as previously described (Wu et al., 2020). *P* < 0.05 was considered indicative of statistical significance.

## Results

### CircRNA expression profiles were significantly altered in intracerebral hemorrhage patients

**FIGURE 1:**
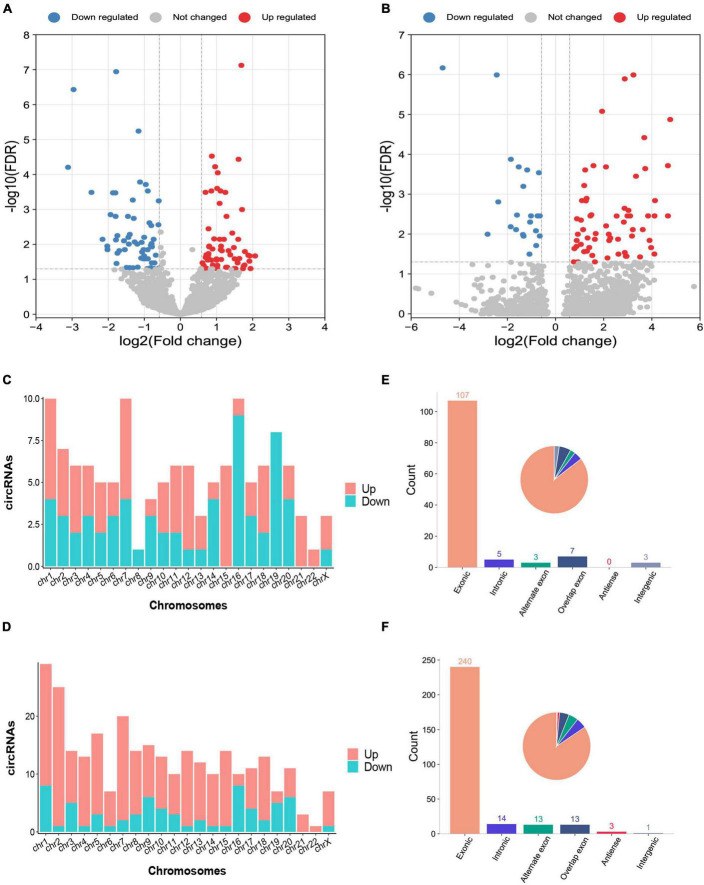
Differentially expressed circRNAs between intracerebral hemorrhage (ICH) patients and healthy controls in the discovery and validation cohorts. **(A,B)** The volcano plot of circRNA expression profiles in ICH patients and controls (fold change ≥ 1.5 and FDR < 0.05) in the discovery (*n* = 44 vs. 31) **(A)** and validation (*n* = 20 vs. 19) **(B)** cohorts. Red dots represent upregulated genes, and blue dots represent downregulated genes. **(C)** The bar diagram shows the circRNA distribution in the chromosomes between 44 ICH patients and 31 healthy controls in the discovery cohort. The red columns represent upregulated circRNAs, while blue columns represent downregulated circRNAs. **(D)** The bar diagram shows the circRNA distribution in the chromosomes between 20 ICH patients and 19 healthy controls in the validation cohort. The red columns represent upregulated circRNAs, while blue columns represent downregulated circRNAs. **(E)** The bar diagram and pie chart show the differentially expressed circRNA distribution in the chromosome region (exonic, intronic, intergenic, alternate exon, overlapping exon and antisense) in 44 ICH patients compared with 31 healthy controls in the discovery cohort. **(F)** The bar diagram and pie chart show the differentially expressed circRNA distribution in the chromosome region (exonic, intronic, intergenic, alternate exon, overlapping exon and antisense) in 20 ICH patients compared with 19 healthy controls in the validation cohort.

The characteristics and demographics of the cohorts of ICH patients, IS patients and healthy controls are shown in Table 1. In RNA sequencing, the significantly differentially expressed circRNAs were determined by a fold change > 1.5 and FDR < 0.05 by DESeq2 methods. In total, 125 circRNAs were significantly altered between patients with ICH and controls, including 63 upregulated circRNAs and 62 downregulated circRNAs in the discovery cohort (Figure 1A and Supplementary Table 1), and 284 circRNAs were significantly altered between patients with ICH and healthy controls in the validation cohort, including 218 upregulated circRNAs and 66 downregulated circRNAs (Figure 1B and Supplementary Table 2). Additionally, the circRNAs were distributed across all chromosomes in both cohorts (Figures 1C,D). There were 107 circRNAs produced by classic exon back-splicing, 3 alternate exons, 5 introns, 7 overlapping exons, and 3 intergenic circRNAs detected between ICH patients and controls in the discovery cohort (Figure 1E), and 240 circRNAs produced by classic exon back-splicing, 13 alternate exons, 14 introns, 13 overlapping exons, 3 antisense and 1 intergenic circRNA were detected between ICH patients and controls in the validation cohort (Figure 1F). Moreover, we observed that 302 and 395 circRNAs were significantly altered between ICH and IS patients in the discovery and validation cohorts, respectively (Supplementary Figures 1A,B).

**TABLE 1:** Demographics and characteristics of the discovery and validation cohorts.

	Discovery cohort				Validation cohort			
				
	Control (*n* = 31)	ICH (*n* = 44)	IS (*n* = 43)	*P*-value	Control (*n* = 19)	ICH (*n* = 20)	IS (*n* = 16)	*P*-value
Age, y	58.9 ± 5.3	55.9 ± 7.2	57.4 ± 5.5	0.09	57.2 ± 7.0	56.7 ± 7.1	57.2 ± 7.7	0.86
Men,%	17 (54.8)	24 (54.5)	21 (48.8)	0.83	10 (52.6)	10 (50)	8 (50)	0.98
BMI, kg/m^2^	24.8 ± 2.9	26.1 ± 6.6	27.6 ± 6.9	0.09	24.9 ± 2.4	25.8 ± 6.8	25.0 ± 2.6	0.90
SBP, mmHg	125.7 ± 10.1	137.4 ± 17.6	138.6 ± 13.6	<0.001	120.3 ± 9.7	171.2 ± 25.7	150.6 ± 19.4	<0.001
DBP, mmHg	79.2 ± 4.3	87.9 ± 10.7	91.8 ± 16.6	<0.001	77.6 ± 9.1	103.7 ± 13.3	89.3 ± 13.9	<0.001
HDL-C, mmol/L	1.4 ± 0.3	1.1 ± 0.3	1.1 ± 0.2	<0.001	1.3 ± 0.3	0.9 ± 0.5	1.0 ± 0.3	0.007
LDL-C, mmol/L	2.9 ± 0.7	2.4 ± 0.8	2.3 ± 0.8	<0.001	2.9 ± 0.9	2.8 ± 0.8	2.7 ± 0.9	0.82
TC, mmol/L	5.5 ± 1.0	4.5 ± 1.0	4.5 ± 1.0	<0.001	4.5 ± 1.0	4.3 ± 0.9	4.9 ± 1.3	0.23
TG, mmol/L	1.4 ± 0.8	1.5 ± 0.9	1.6 ± 0.6	0.41	1.2 ± 0.5	1.4 ± 0.6	2.3 ± 1.5	0.004
GLU, mmol/L	6.0 ± 1.8	6.3 ± 1.6	5.9 ± 1.3	0.17	5.3 ± 0.6	5.5 ± 1.7	6.0 ± 1.1	0.21
Smoking,%				0.95				0.92
Never	19 (61.3)	28 (63.7)	26 (60.5)		13 (68.4)	14 (70)	11 (68.7)	
Former	4 (12.9)	5 (13.6)	8 (18.6)		3 (15.8)	2 (10)	2 (12.5)	
Current	8 (25.8)	11 (22.7)	9 (20.9)		3 (15.8)	4 (20)	3 (18.8)	
Drinking,%				0.98				0.96
Non-drinker	20 (64.5)	28 (63.6)	27 (62.8)		11 (57.9)	12 (60)	10 (62.5)	
Drinker	11 (35.5)	16 (36.4)	16 (37.2)		8 (42.1)	8 (40)	6 (37.5)	

Data are expressed as the mean ± standard deviation or n (%).

BMI, Body mass index; SBP, Systolic blood pressure; DBP, Diastolic blood pressure; TC, Total cholesterol; TG, Triacylglycerol; HDL-C, High-density lipoprotein cholesterol; LDL-C, Low-density lipoprotein cholesterol; GLU, Glucose; ICH, Intracerebral hemorrhage; IS, ischemic stroke.

Statistical comparisons for percentages were performed using the chi-square test. Comparisons between means or medians were performed using one-way ANOVA.

### Gene ontology enrichment and kyoto encyclopedia of genes and genomes pathway analyses of circRNA host genes

**FIGURE 2:**
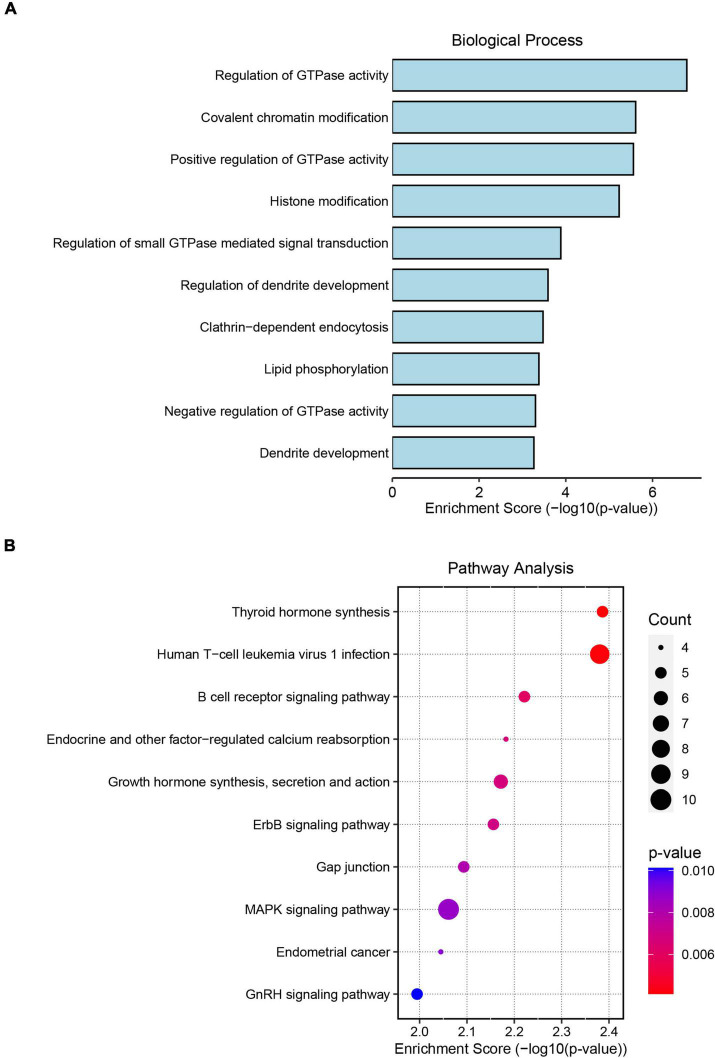
Gene Ontology (GO) and Kyoto Encyclopedia of Genes and Genomes (KEGG) pathway analyses of significantly altered circRNA host genes. **(A)** The top 10 biological process terms from GO enrichment analysis of differentially expressed circRNA host genes. **(B)** The top 10 KEGG pathway analyses of differentially expressed circRNA host genes.

To assess the potential regulatory mechanism of differentially expressed circRNAs in host gene transcription after ICH, we performed GO and KEGG pathway analyses of the host genes of the altered circRNAs in the two cohorts. The top GO terms in the biological process category indicated that the host genes were involved in the regulation of GTPase activity, covalent chromatin modification, histone modification, regulation of dendrite development and lipid phosphorylation (Figure 2A). KEGG pathway analysis showed that the host genes were mainly involved in the MAPK signaling network, B-cell receptor signaling, ERBB receptor signaling network, thyroid hormone synthesis and lysine degradation (Figure 2B).

### Consistently altered circRNAs in the discovery and validation cohorts

**FIGURE 3:**
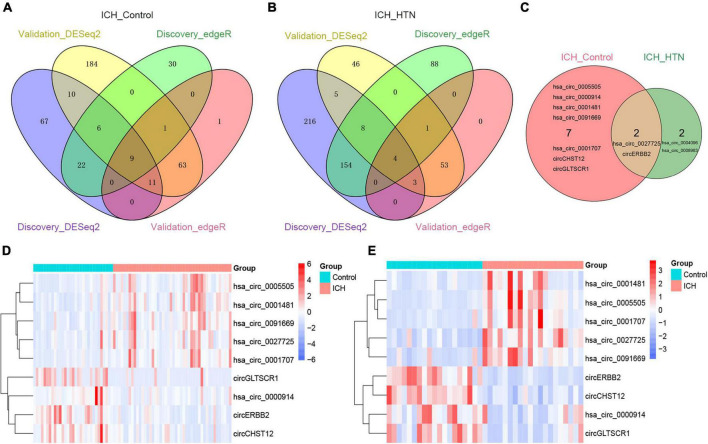
Consistently differentially expressed circRNAs between intracerebral hemorrhage (ICH) and controls or hypertension (HTN) in the discovery and validation cohorts by DESeq2 and edgeR methods. **(A)** Venn diagram showing consistently altered circRNAs (fold change ≥ 1.5 and FDR < 0.05) in ICH patients compared with controls in the discovery (*n* = 44 vs. 31) and validation cohorts (*n* = 20 vs. 19) with both the DESeq2 and edgeR methods. **(B)** Venn diagram showing consistently altered circRNAs (fold change ≥ 1.5 and FDR < 0.05) in ICH compared with HTN in the discovery (*n* = 44 vs. 42) and validation cohorts (*n* = 20 vs. 18) with both the DESeq2 and edgeR methods. **(C)** Venn diagram showing the common altered circRNAs (fold change ≥ 1.5 and FDR < 0.05) in the ICH patients compared with healthy controls and ICH compared with HTN in both cohorts. Hierarchical clustering of nine consistently differentially expressed circRNAs between ICH patients and healthy controls in the discovery (*n* = 44 vs. 31) **(D)** and validation (*n* = 20 vs. 19) **(E)** cohorts. Blue represents downregulated circRNAs, red represents upregulated circRNAs, and white represents no changes in circRNA expression. The column represents a sample, and each row represents a single circRNA. The red color label represents the ICH group, and the green color label represents the healthy control group. The label color scales indicate the circRNA relative expression levels in the ICH and control groups.

To elucidate the underlying mechanism by which the circRNAs affected ICH more specifically, we screened the common circRNAs in the two cohorts by both DESeq2 and edgeR methods (Supplementary Tables 1–4) and found that 9 circRNAs overlapped between the ICH patients and controls (Figure 3A). Similarly, there were 4 consistent circRNAs between ICH and hypertension (HTN) in our previous study (Figure 3B) (Bai et al., 2021); 2 of them were consistently altered in the two comparison groups, including hsa_circ_0027725 and a novel circRNA (host gene *ERBB2*) we named circERBB2 (Figure 3C).

The nine consistently altered circRNAs included five upregulated circRNAs and four downregulated circRNAs. The five upregulated circRNAs in ICH were hsa_circ_0001707, hsa_circ_0091669, hsa_circ_0005505, hsa_circ_0001481 and hsa_circ_0027725; the 4 downregulated circRNAs in ICH were hsa_circ_0000914 and three novel circRNAs that we named according to their host genes, circCHST12 (host gene *CHST12*), circERBB2 and circGLTSCR1 (host gene *GLTSCR1*) (Table 2). The 9 circRNA expression variants are shown with hierarchical clustering heatmaps in the discovery and validation cohorts (Figures 3D,E), which indicated that the circRNA expression profiles in ICH patients were distinct from those in healthy control groups.

**TABLE 2:** The consistently altered circRNAs in intracerebral hemorrhage (ICH) patients compared with controls.

Location	circRNA ID	Discovery		Validation		Up/Down	Host gene	Type
						
		FC	FDR	FC	FDR			
chr7:48541721-48542148: +	hsa_circ_0001707	3.038	2.4E-04	3.817	2.1E-09	up	Null	Intronic
chrX:147733519-147744289: +	hsa_circ_0091669	1.827	0.005	2.138	1.4E-06	up	AFF2	Exonic
chr12:66597490-66622150: +	hsa_circ_0005505	2.903	0.007	7.329	2.8E-10	up	IRAK3	Exonic
chr5:49694940-49707217: –	hsa_circ_0001481	1.850	0.012	2.803	3.9E-06	up	EMB	Exonic
chr12:94562928-94580249: +	hsa_circ_0027725	2.179	0.037	2.281	4.9E-07	up	PLXNC1	Exonic
chr7:2477438-2483381: +	circCHST12	0.334	0.007	0.395	5.5E-07	down	CHST12	Alternate exon
chr19:18648410-18649246: –	hsa_circ_0000914	0.510	0.010	0.595	4.6E-06	down	FKBP8	Exonic
chr17:37866065-37872192: +	circERBB2	0.278	0.020	0.184	1.7E-10	down	ERBB2	Exonic
chr19:48185232-48198731: +	circGLTSCR1	0.183	0.037	0.275	1.3E-05	down	GLTSCR1	Exonic

FC, fold change; FDR, false discovery rate.

Likewise, we detected 20 consistent circRNAs between ICH and IS patients in the two cohorts by both DESeq2 and edgeR methods (Supplementary Figure 1C). Notably, 3 circRNAs were in the intersection between ICH versus controls (9 consistent circRNAs) and ICH versus IS (20 consistent circRNAs), including circERBB2, circCHST12 and hsa_circ_0005505 (Supplementary Figure 1D).

### Investigation of the nine circRNAs as independent predictors of intracerebral hemorrhage

To further explore the potential value of candidate circRNAs as ICH biomarkers, logistic regression models were performed to identify whether nine circRNAs could be predictors of ICH occurrence. As shown in Table 3, after adjusting for age, sex, body mass index (BMI), systolic blood pressure (SBP), diastolic blood pressure (DBP), total cholesterol (TC), triacylglycerol (TG), high-density lipoprotein cholesterol (HDL-C), low-density lipoprotein cholesterol (LDL-C), smoking and alcohol consumption, per unit of increase in hsa_circ_0001707, hsa_circ_0091669, hsa_circ_0005505, hsa_circ_0001481 and hsa_circ_0027725, the odds ratios for ICH occurrence were 2.23 (95% CI: 1.294–3.842; *P* = 0.004), 3.372 (95% CI: 1.665–6.867; *P* = 0.001), 2.216 (95% CI: 1.363–3.316; *P* = 0.001), 4.750 (95% CI: 2.054–10.985; *P* < 0.001) and 2.156 (95% CI: 1.170–3.974; *P* = 0.014), respectively. In addition, the adjusted ORs were 0.009 (95% CI: 0.001–0.097; *P* < 0.001), 0.160 (95% CI: 0.051–0.507; *P* = 0.002), 0.019 (95% CI: 0.002–0.157; *P* < 0.001) and 0.122 (95% CI: 0.037–0.410; *P* = 0.001) per unit increase in circCHST12, hsa_circ_0000914, circERBB2 and circGLTSCR1, respectively.

**TABLE 3:** Logistic regression analysis to identify circRNAs as independent predictive factors of intracerebral hemorrhage (ICH).

circRNA ID	Adjusted risk factors			Up/Down	Host gene
			
	OR	95% CI	*P*-value		
hsa_circ_0001707	2.230	1.294–3.842	0.004	up	Null
hsa_circ_0091669	3.372	1.655–6.867	0.001	up	AFF2
hsa_circ_0005505	2.216	1.363–3.316	0.001	up	IRAK3
hsa_circ_0001481	4.750	2.054–10.985	<0.001	up	EMB
hsa_circ_0027725	2.156	1.170–3.974	0.014	up	PLXNC1
circCHST12	0.009	0.001–0.097	<0.001	down	CHST12
hsa_circ_0000914	0.160	0.051–0.507	0.002	down	FKBP8
circERBB2	0.019	0.002–0.157	<0.001	down	ERBB2
circGLTSCR1	0.122	0.037–0.041	0.001	down	GLTSCR1

Risk factors included SBP, systolic blood pressure; DBP, diastolic blood pressure; TG, triacylglycerol; TC, total cholesterol; LDL-C, low-density lipoprotein cholesterol; HDL-C, high-density lipoprotein cholesterol, smoking and alcohol consumption; ICH, intracerebral hemorrhage; OR, odds ratio; CI, confidence interval.

### Validation of the differentially expressed circRNAs by quantitative real-time polymerase chain reaction

**FIGURE 4:**
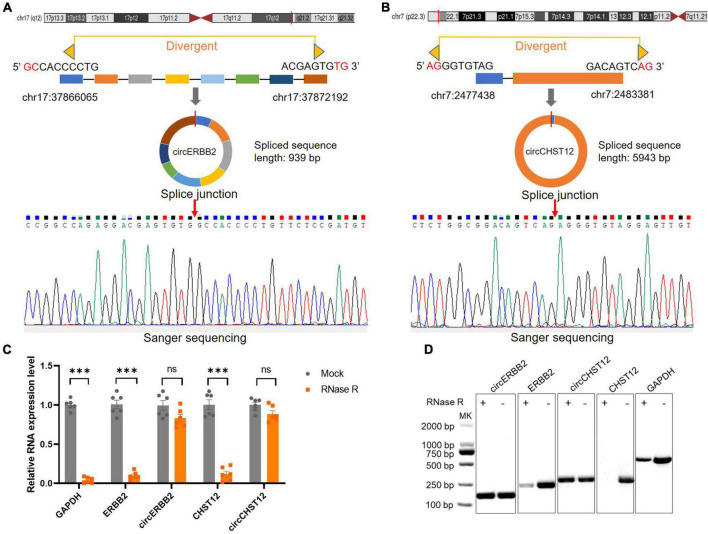
Identification of novel circular RNAs circERBB2 and circCHST12. **(A,B)** Schematic diagrams and Sanger sequencing illustrated the back-splice junction site of circERBB2 **(A)** and circCHST12 **(B)**. **(C)** RT–qPCR showed the expression of *GAPDH*, *ERBB2*, *circERBB2*, *CHST12* and *circCHST12* administered RNase R or mock control (*n* = 6 per group). **(D)** Representative agarose gel pictures showing the relative expression of *GAPDH*, *ERBB2*, *circERBB2*, *CHST12*, and *circCHST12* administered RNase R or mock control. Data are presented as the mean ± standard deviation. ^***^ *p* < 0.001. ns: no significant. Statistical significance was assessed using unpaired two-tailed Student’s *t*-test.

To verify the novel circRNAs circERBB2 and circCHST12 are really circular form, we first blasted the sequences and confirmed the back-splice junction sites and assayed them by RT–PCR with divergent primers. Next, Sanger sequencing was performed to illustrate the junction site. The results showed that circERBB2, located at chr17:37866065-37872192 (genomic length: 6127 bp, spliced sequence length: 939 bp), was derived from exons 9–16 of the *ERBB2* gene (Figure 4A). circCHST12, located at chr7:2477438-2483381 (genomic length: 5943 bp, spliced sequence length: 5943 bp), was derived from exon 1 and partial exon 2 of the *CHST12* gene (Figure 4B). RT–qPCR analysis of total RNA after RNase R or control treatment indicated that circERBB2 and circCHST12 were resistant, while *ERBB2*, *CHST12* and *GAPDH* mRNA transcripts were degraded (Figures 4C,D). These data established that circERBB2 and circCHST12 are two *bona fide* circRNAs.

**FIGURE 5:**
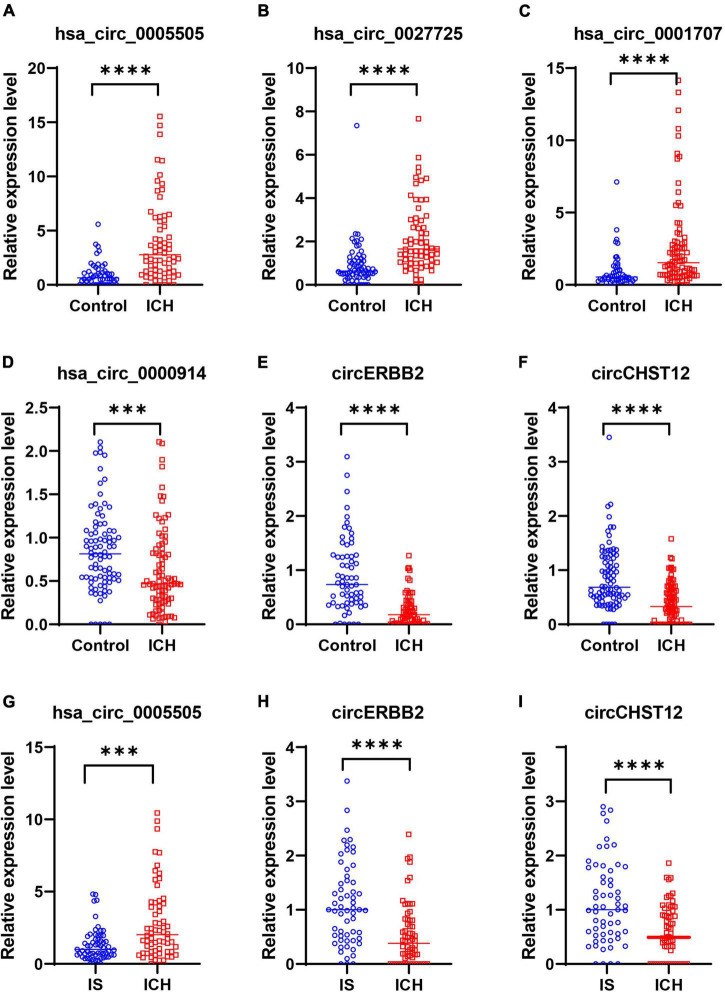
Validation of circRNA expression levels by quantitative real-time polymerase chain reaction (RT–qPCR). **(A–F)** RT–qPCR results validated the expression levels of candidate circRNAs in all samples between 64 intracerebral hemorrhage (ICH) patients and 50 healthy controls. **(A)** hsa_circ_0005505, **(B)** hsa_circ_0027725, **(C)** hsa_circ_0001707, **(D)** hsa_circ_0000914, **(E)** circERBB2 and **(F)** circCHST12. **(G–I)** RT–qPCR results validated the expression levels of hsa_circ_0005505 **(G)**, circERBB2 **(H)** and circCHST12 **(I)** between 64 ICH patients and 59 ischemic stroke (IS) patients. The data are presented as the median (interquartile range). ^***^p < 0.001, ^*⁣*⁣**^p < 0.0001. Statistical significance was assessed using the Mann–Whitney U test.

Next, to confirm the expression of circRNAs in the high-throughput results, we selected three upregulated circRNAs (hsa_circ_0001707, hsa_circ_0005505 and hsa_circ_0027725) and three downregulated circRNAs (hsa_circ_0000914, circERBB2 and circCHST12) of the above consistently altered circRNAs for further validation by RT–qPCR in all samples. The expression levels of these circRNAs were consistent with the RNA sequencing results, including three upregulated circRNAs and three downregulated circRNAs that were significantly altered in patients with ICH compared with control subjects (Figures 5A–F). Moreover, the expression levels of circERBB2, circCHST12 and hsa_circ_0005505 were also significantly altered between ICH and IS patients (Figures 5G–I). These results were consistent with the levels obtained by RNA sequencing, supporting the accuracy and reliability of the data.

### Performance evaluation of the candidate circRNAs with classification algorithms

To evaluate applicable biomarkers for ICH, we used mutual information (MI) and random forest (RF) algorithms to screen circRNA marker signatures according to the expression levels in all samples. We obtained the signature of the top 10 circRNAs in the two algorithms and found 4 circRNAs [hsa_circ_0005806, circERBB2, circCHST12, circFBRS (host gene *FBRS*)] in the intersection (Supplementary Table 5). However, there was no significant difference in hsa_circ_0005806 or circFBRS expression levels between the ICH patients and controls in the validation cohort (Supplementary Figure 2). Finally, we focused on evaluating the diagnostic value of circERBB2 and circCHST12 as potential ICH biomarkers in further statistical analysis.

**FIGURE 6:**
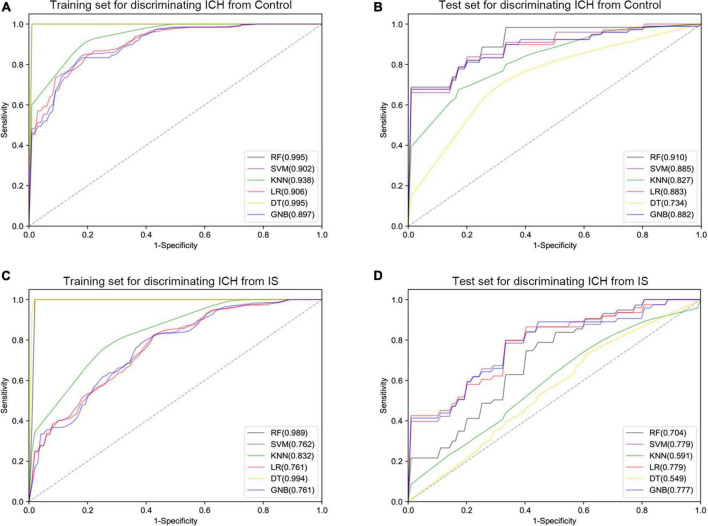
Receiver operating curve (ROC) plot of the six classifier performances based on AUC in the training set and test set. **(A,B)** ROC plot of the six classifier performances based on AUC in the training set **(A)** and test set **(B)** for discriminating intracerebral hemorrhage (ICH) from healthy controls. **(C,D)** ROC plot of the six classifier performances based on AUC in the training set **(C)** and test set **(D)** for discriminating ICH from ischemic stroke (IS) patients. SVM, support vector machine; RF, random forest; KNN, K-nearest neighbor; LR, logistic regression; DT, decision tree; GNB, Gaussian naive Bayes.

Furthermore, six different classifier algorithms were executed to assess the validity of the candidate circRNAs. By using 10-fold cross-validation, the average performance measurement values of the candidate circRNAs in ICH were computed and are summarized in Table 4. The six machine learning classifiers based on test accuracies and AUCs in the training set and validation set are presented in Figure 6. The RF provides greater accuracy values of 0.995 and 0.910 than the other five classifiers in the training and test sets between ICH and controls, respectively (Figures 6A,B). We also evaluated the performance of the circERBB2 and circCHST12 signatures for discriminating ICH from IS patients and observed that the RF had the highest value of 0.989 in the training set and the SVM had the highest value of 0.779 in the test set (Figures 6C,D and Supplementary Table 6). These results indicate that the combination of the circERBB2 and circCHST12 signatures is capable of identifying ICH with high accuracy according to expression levels.

**TABLE 4:** Classification performance for the two-circRNA signatures between intracerebral hemorrhage (ICH) patients.

	Sensitivity (%)	Specificity (%)	Accuracy (%)	PPV (%)	NPV (%)	AUC
**RF**						
Training set	100	100	100	100	100	0.995(0.983–1)
Test set	80.83	74.81	78.48	80.10	77.48	0.910(0.857–0.963)
**KNN**						
Training set	91.71	81.32	87.26	86.66	88.33	0.938(0.894–0.982)
Test set	80.56	69.63	76.89	78.08	72.62	0.827(0.753–0.901)
**DT**						
Training set	100	100	100	100	100	0.995(0.983–1)
Test set	77.03	69.81	73.26	78.21	66.48	0.734(0.644–0.824)
**LR**						
Training set	86.35	76.82	82.28	83.16	80.99	0.906(0.852–0.960)
Test set	84.03	72.64	80.38	80.62	76.33	0.883(0.822–0.944)
**GNB**						
Training set	90.90	65.11	79.79	77.48	84.41	0.897(0.840–0.954)
Test set	90.14	63.98	79.62	77.46	82.17	0.882(0.821–0.943)
**SVM**						
Training set	93.25	64.25	80.75	77.51	87.90	0.902(0.846–0.957)
Test set	88.89	63.98	79.62	76.78	85.83	0.885(0.825–0.945)

ICH, intracerebral hemorrhage; RF, random forest; KNN, K-nearest neighbor; LR, logistic regression; DT, decision tree; GNB, Gaussian naive Bayes; SVM, support vector machine; PPV, positive predictive value; NPV, negative predictive value; AUC, area under the curve.

### Correlation of the circERBB2 and circCHST12 expression levels with clinical characteristics

Additionally, we performed Spearman’s correlation analysis to test the correlation of the expression levels of circCHST12 and circERBB2 with ICH patient clinical characteristics. The results showed that the circERBB2 expression levels positively correlated with HDL-C and negatively correlated with SBP, DBP and alcohol consumption in ICH patients (*P* < 0.05); the circCHST12 expression levels positively correlated with LDL-C and negatively correlated with SBP, DBP, glucose, white blood cells and alcohol consumption (*P* < 0.05) (Table 5). These results indicated that circERBB2 and circCHST12 may be involved in the pathogenesis of ICH.

**TABLE 5:** Correlation between baseline characteristic and circRNA levels in intracerebral hemorrhage (ICH) patients.

Parameters	circERBB2		circCHST12	
		
	Coefficient	*P*-value	Coefficient	*P*-value
Age, y	0.143	0.128	–0.018	0.850
Sex (male)	0.017	0.895	0.022	0.814
BMI, kg/m^2^	0.044	0.646	0.103	0.274
SBP, mmHg	–0.373	< 0.001*	–0.240	0.010*
DBP, mmHg	–0.418	< 0.001*	–0.309	0.001*
HDL-C, mmol/L	0.190	0.043*	0.153	0.104
LDL-C, mmol/L	0.157	0.096	0.224	0.016*
TC, mmol/L	0.165	0.079	0.016	0.419
TG, mmol/L	–0.085	0.367	–0.164	0.182
GLU, mmol/L	–0.06	0.525	–0.273	0.003*
UA, μmol/L	0.193	0.097	0.218	0.060
TBIL, μmol/L	–0.023	0.846	0.001	0.992
BUN, mmol/L	–0.027	0.817	0.094	0.442
WBC, 10^9^/L	–0.283	0.014*	–0.366	0.001*
Smoking	–0.063	0.504	–0.153	0.104
Alcohol consumption	–0.215	0.022*	–0.307	0.001*

ICH, Intracerebral hemorrhage; BMI, Body mass index; SBP, Systolic blood pressure; DBP, Diastolic blood pressure; TC, Total cholesterol; TG, Triacylglycerol; HDL-C, High-density lipoprotein cholesterol; LDL-C, Low-density lipoprotein cholesterol; GLU, Glucose; UA, Uric acid; TBIL, Total bilirubin; BUN, Blood urea nitrogen; WBC, White blood cell. *p < 0.05.

### Evaluation of the diagnostic value of circERBB2 and circCHST12 in intracerebral hemorrhage patients

**FIGURE 7:**
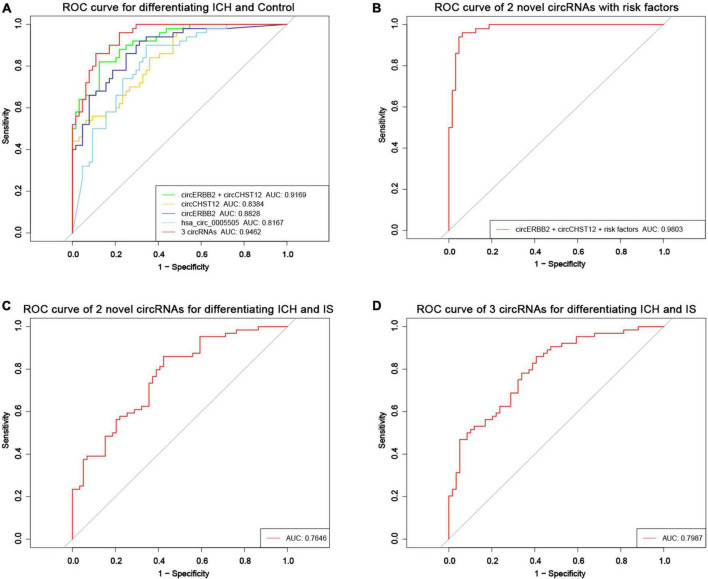
Evaluation of the circRNA diagnostic value in ICH patients. **(A)** Receiver operating characteristic (ROC) curves were calculated using the expression levels of circERBB2, circCHST12 and hsa_circ_0005505 for differentiating patients with intracerebral hemorrhage (ICH) and healthy controls (*n* = 64 vs. 50). **(B)** ROC curves of combining circERBB2 and circCHST12 with ICH risk factors to differentiate patients with ICH and healthy controls in all samples (*n* = 64 vs. 50). **(C)** ROC curves of combining circERBB2 and circCHST12 for differentiating patients with ICH and IS patients in all samples (*n* = 64 vs. 59). **(D)** ROC curves of two novel circRNAs, circERBB2 and circCHST12, combined with hsa_circ_0005505 for differentiating patients with ICH and IS patients in all samples (*n* = 64 vs. 59).

**FIGURE 8:**
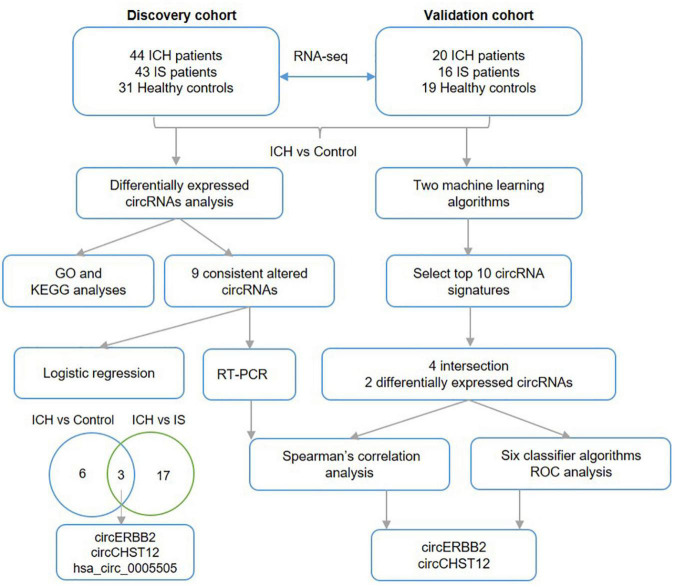
Work flow. The diagram of the data analysis process in this study.

Receiver operating curve (ROC) analysis was performed to explore the potential diagnostic value of circERBB2 and circCHST12. The signatures of circERBB2 for differentiating between patients with ICH and healthy control subjects showed an AUC of 0.883 (95% CI: 0.811–0.937) with a sensitivity of 68.2% and a specificity of 92%; the signatures of circCHST12 showed an AUC of 0.838 (95% CI: 0.769–0.908) with a sensitivity of 93% and a specificity of 71.6% (Figure 7A). The combination of circERBB2 and circCHST12 for differentiating between patients with ICH and healthy controls showed an AUC of 0.917 (95% CI: 0.869–0.965), with a sensitivity of 87.5% and a specificity of 82% (Figure 7A). We next performed a multifactor risk logistic regression model, the combination of circERBB2 and circCHST12 together with the risk factors (age, sex, BMI, SBP, DBP, TC, TG, HDL-C, LDL-C, smoking and alcohol consumption) showed that the AUC was increased to 0.980 (95% CI: 0.959–1), the sensitivity was 93.8%, and the specificity was 96% (Figure 7B). The addition of circERBB2 and circCHST12 to the previously known risk factors improved the predictive ability, with an NRI of 20.3% and IDI of 23.7% (*P* < 0.001). The AUC of circERBB2 and circCHST12 for differentiating between ICH and IS patients was 0.765 (95% CI: 0.682–0.847); the sensitivity was 57.6%, and the specificity was 85.9% (Figure 7C).

hsa_circ_0005505 was upregulated in both ICH compared with controls and ICH compared IS patients. Furthermore, we evaluated the diagnostic values of the two novel circRNA combinations of hsa_circ_0005505 for identifying ICH. The combination of hsa_circ_0005505, circERBB2 and circCHST12 for differentiating between patients with ICH and healthy controls showed an AUC of 0.946 (95% CI: 0.910–0.982), with a sensitivity of 89.1% and a specificity of 86% (Figure 7A); the AUC was 0.799 (95% CI: 0.722–0.875), with a sensitivity of 59.3% and a specificity of 89.5% for differentiating between patients with ICH and IS patients (Figure 7D). These results indicate that hsa_circ_0005505, novel circERBB2 and circCHST12, individually or combined, serve as potential diagnostic biomarkers for identifying ICH (Figure 8).

## Discussion

In the present study, we first investigated the circRNA profiles in the peripheral blood of ICH patients and healthy controls by using RNA sequencing in two independent cohorts. Functional analysis indicated that the differentially expressed circRNAs are involved in many pathophysiologic processes of ICH. By using two independent analysis strategies, we obtained nine circRNAs that were consistently altered in both cohorts, including five upregulated circRNAs and four downregulated circRNAs. Furthermore, based on machine learning classification, we screened two candidates, circERBB2 and circCHST12, to explore their diagnostic value as potential biomarkers in ICH patients. The AUC was 0.917 (95% CI: 0.869–0.965), with a sensitivity of 87.5% and a specificity of 82% for distinguishing between ICH patients and healthy controls. In combination with ICH risk factors, the AUC was 0.980 (95% CI: 0.959–1), sensitivity was 93.8% and specificity was 96% in ICH diagnosis. Moreover, logistic regression analysis and Spearman’s correlation test demonstrated that downregulation of circERBB2 and circCHST12 may be independent risk factors for ICH. Additionally, the expression level of circERBB2 correlated with SBP and HDL-C; circCHST12 expression levels correlated with LDL-C, SBP, DBP and white blood cells, indicating that circERBB2 and circCHST12 might be heavily involved in the pathology of ICH. Our data show that circERBB2 and circCHST12 may be novel biomarkers for ICH diagnosis. Together with hsa_circ_0005505, circERBB2 and circCHST12 showed high accuracy for identifying ICH. A previous study revealed that hsa_circ_0005505 was upregulated in ruptured intracranial aneurysm tissues, promoted proliferation and migration and suppressed apoptosis of vascular smooth muscle cells *in vitro* (Chen X. et al., 2021), indicating that hsa_circ_0005505 may be associated with the pathological process of cerebrovascular diseases.

Intracerebral hemorrhage (ICH) is a multifactorial disease with high incidence and mortality that imposes a large socioeconomic burden. Identifying novel potential biomarkers for the early diagnosis of ICH would be part of risk prediction. CircRNAs are produced by host gene back-splicing, and closed RNAs without a free 3′ or 5′ end are resistant to exonuclease digestion (Jeck and Sharpless, 2014), which makes them more stable and better biomarkers of human disease. Furthermore, circRNAs are highly expressed in many tissues, particularly the human brain, and in blood (Patop et al., 2019). There is growing evidence that the circRNA expression profile is altered in IS (Dong et al., 2020; Ostolaza et al., 2020; Zuo et al., 2020; Liu Y. et al., 2022), indicating that circRNAs have the potential to serve as biomarkers and therapeutic targets in IS. Moreover, the circRNA expression profiles were altered in rat brain tissues after ICH (Zhong et al., 2020; Bai et al., 2021). However, the changes in circRNA expression in the peripheral blood of ICH patients remain unclear. Our previous study demonstrated that hsa_circ_0001240, hsa_circ_0001947 and hsa_circ_0001386 were promising biomarkers for predicting and diagnosing hypertensive ICH (Bai et al., 2021). In this study, we first investigated whether circRNA profiles were significantly altered between ICH patients and healthy controls, which provides new insights into understanding the epigenomic mechanisms of ICH.

In this study, we found that circERBB2 may serve as a novel biomarker in ICH diagnosis. Previous studies have identified blood biomarkers, such as glial fibrillary acid protein (GFAP), retinol binding protein 4 and N-terminal pro B-type natriuretic peptide, that distinguish IS from ICH with moderate accuracy (Bustamante et al., 2021) and metabolic biomarkers for ICH diagnosis (Zhang et al., 2021). The AUCs of S100 and IL6 were 0.65 and 0.59 (Bhatia et al., 2020), respectively, and GFAP had a sensitivity of 78% and a specificity of 95% between ICH and IS (Kumar et al., 2020). ncRNAs have been identified as critical novel regulators of cardiovascular risk factors and cell functions and are thus important candidates to improve diagnostics and prognosis assessment (Poller et al., 2018). In the present study, we identified that the AUC of circERBB2 was 0.883 for distinguishing between ICH patients and healthy controls, with a sensitivity and specificity of 68.2% and 92%, respectively. The signatures of circCHST12 showed an AUC of 0.838 with a sensitivity of 93% and a specificity of 71.6%. The combination of circERBB2 and circCHST12 with ICH risk factors increased the predictive value for the identification of ICH. These findings were better than the diagnostic value of three previously identified circRNAs [hsa_circ_0001240 (AUC = 0.808), hsa_circ_0001947 (AUC = 0.798) and hsa_circ_0001386 (AUC = 0.806)] in ICH (Bai et al., 2021). Additionally, we observed that downregulation of circERBB2 was positively associated with HDL-C and negatively correlated with SBP and DBP. Lowering blood lipids was associated with an increased risk of ICH (Sun et al., 2019), and high blood pressure was found to be the most prevalent stroke risk factor (Feigin et al., 2016; Wang et al., 2017). Thus, we speculate that a decrease in circERBB2 expression levels might correlate with an increased risk of ICH occurrence. These findings indicate that circERBB2 might play vital roles in the pathogenesis and pathology of ICH.

The protein ERBB2 is a member of a family of epidermal growth factor receptors that are involved in aberrant signaling and cell migration, growth, adhesion, and differentiation (Strickler et al., 2022). A previous study demonstrated that circERBB2 (chr17: 39,708,320–39,710,481; length: 676 bp) serves as an important regulator of cancer cell proliferation and has the potential to be a new therapeutic target for gallbladder cancer (Huang et al., 2019) and breast cancer (Huang Y. et al., 2021). Our study identified circERBB2 (chr17: 37,866,065–37,872,192; genomic length: 6127 bp, spliced sequence length: 939 bp), which is a novel back-splicing circRNA that has never been reported thus far, at a different chromosomal position. Carbohydrate sulfotransferases (CHSTs) are a class of key enzymes that contribute to tissue remodeling. CHST12 is a significant member of the CHST family, and a previous study demonstrated that CHST12 may be a novel biomarker for glioblastoma; it regulates cell proliferation and mobility via the WNT/β-catenin pathway (Wang et al., 2021). One study reported that hsa_circ_0134005 (chr7:2472197-2477555; genomic length: 5358 bp, spliced sequence length: 5358 bp) is derived from the CHST12 gene (Rybak-Wolf et al., 2015). This study identified circCHST12 (chr7:2477438-2483381; genomic length: 5943 bp, spliced sequence length: 5943 bp) derived from exon 1 and partial exon 2 of the CHST12 gene, which is a novel back-splicing circRNA that has never been reported thus far at a different chromosomal position.

CircRNAs are involved in the translational and transcriptional regulation of the pathological mechanisms of many disorders (Shan et al., 2017; Aufiero et al., 2019). CircRNAs can act as miRNA sponges and are expected to influence downstream miRNA function, further regulating the expression levels of target mRNAs (Hansen et al., 2013). We performed GO and KEGG analyses to investigate the enrichment of differentially expressed circRNAs. Functional analysis demonstrated that the circRNA host genes were mainly involved in GTPase activity, covalent chromatin modification, histone modification, the MAPK signaling pathway and the ERBB signaling pathway. Activation of the MAPK signaling pathway is involved in the progression of injury following ICH (Ding et al., 2020; Guo et al., 2020). Recently, research identified that knockdown of circERBB2 suppressed the PDGF-BB-induced proliferation, migration, and inflammatory response of human airway smooth muscle cells via miR-98-5p/IGF1R signaling (Huang J. Q. et al., 2021). The phenotype of smooth muscle cells transforming from a contractile to a synthetic phenotype plays an essential role in the onset of brain vascular pathological progression (Bennett et al., 2016; Rho et al., 2017). In this study, we speculated that the downregulation of the novel circERBB2 in ICH patients might contribute to the pathogenesis of ICH via the phenotype of smooth muscle cell transformation.

Notably, there are some limitations of this study. First, we should perform a larger multicenter study with more participants to externally validate the candidate biomarkers. Second, further studies should be performed to explore how hsa_circ_0005505, circERBB2 and circCHST12 contribute to the pathogenesis and development of ICH with cell- or animal-based experiments. Additionally, our study lacked follow-up information for ICH patients, and the prognostic value of these candidate circRNAs should be assessed in subsequent studies. We expect that hsa_circ_0005505, circERBB2 and circCHST12 will provide new insights for a better understanding of the pathogenesis of ICH and help to improve the diagnosis and prognostic assessment of ICH in clinical practice.

## Conclusion

In this study, we provided a transcriptome-wide overview of aberrantly expressed circRNAs in the peripheral blood of ICH patients and identified hsa_circ_0005505 and novel circERBB2 and circCHST12 as promising biomarkers for diagnosing ICH based on machine learning algorithms.

## Data availability statement

The datasets presented in this study can be found in online repositories. The names of the repository/repositories and accession number(s) can be found in the article/Supplementary material.

## Ethics statement

The studies involving human participants were reviewed and approved by Human Ethics Committee, Fuwai Hospital (Approval No. 2016-732). The patients/participants provided their written informed consent to participate in this study.

## Author contributions

CB, YS, and LS: design and experiment. XH and FW: data analyses. CB and LZ: manuscript preparation. JL, LY, and JC: manuscript review. All authors contributed to the article and approved the submitted version.

## Footnotes

1
http://www.ensembl.org/index.html

2
https://www.ncbi.nlm.nih.gov/tools/primer-blast/
