# Supplementary material for: Machine learning-based identification of the novel circRNAs circERBB2 and circCHST12 as potential biomarkers of intracerebral hemorrhage
Source: Front Neurosci. 2022 Nov 29;16:1002590. doi: 10.3389/fnins.2022.1002590 (PMC9745062; doi:10.3389/fnins.2022.1002590)
Supplement: Supplementary file 1 [file Data_Sheet_1.docx]

**Supplementary Materials**

1. **Supplementary Figures**


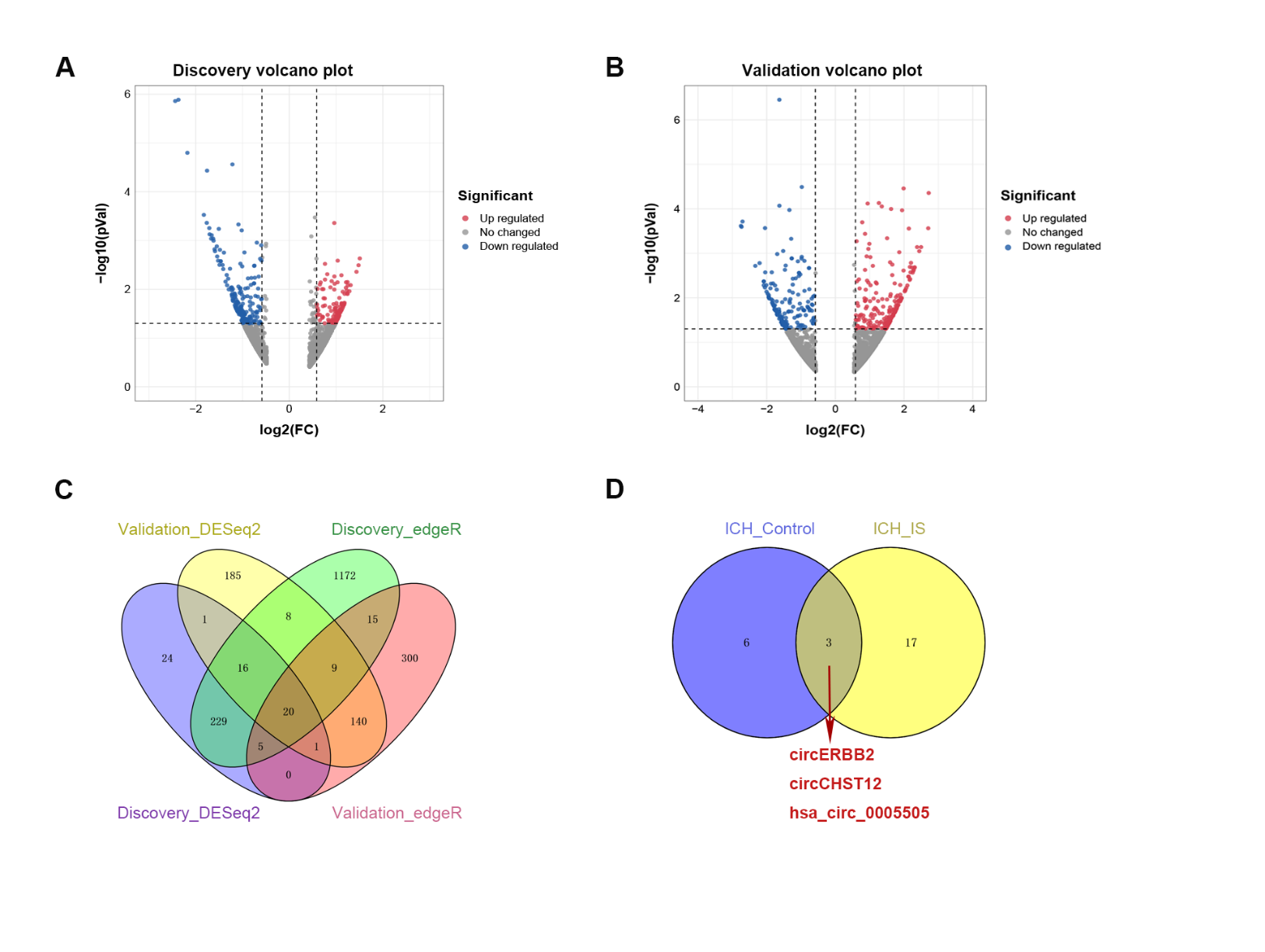


**Supplementary Figure 1. Differentially expressed circular RNAs (circRNAs) between intracerebral hemorrhage (ICH) patients and ischemic stroke (IS) patients in the discovery and validation cohorts.** (**A**) The volcano plot of circRNA expression profiles between 44 ICH patients and 43 IS patients in the discovery cohort with the DESeq2 method. (**B**) The volcano plot of circRNA expression profiles between 20 ICH patients and 16 IS patients in the validation cohort with the DESeq2 method. (**C**) Venn diagram showing consistently altered circRNAs in ICH patients compared with IS patients in the discovery (44 ICH patients vs 43 IS patients) and validation cohorts (20 ICH patients vs 16 IS patients) with both the DESeq2 and edgeR methods. (**D**) Venn diagram showing the common circRNAs in the ICH patients compared with healthy controls and ICH compared with IS patients in both cohorts.

**
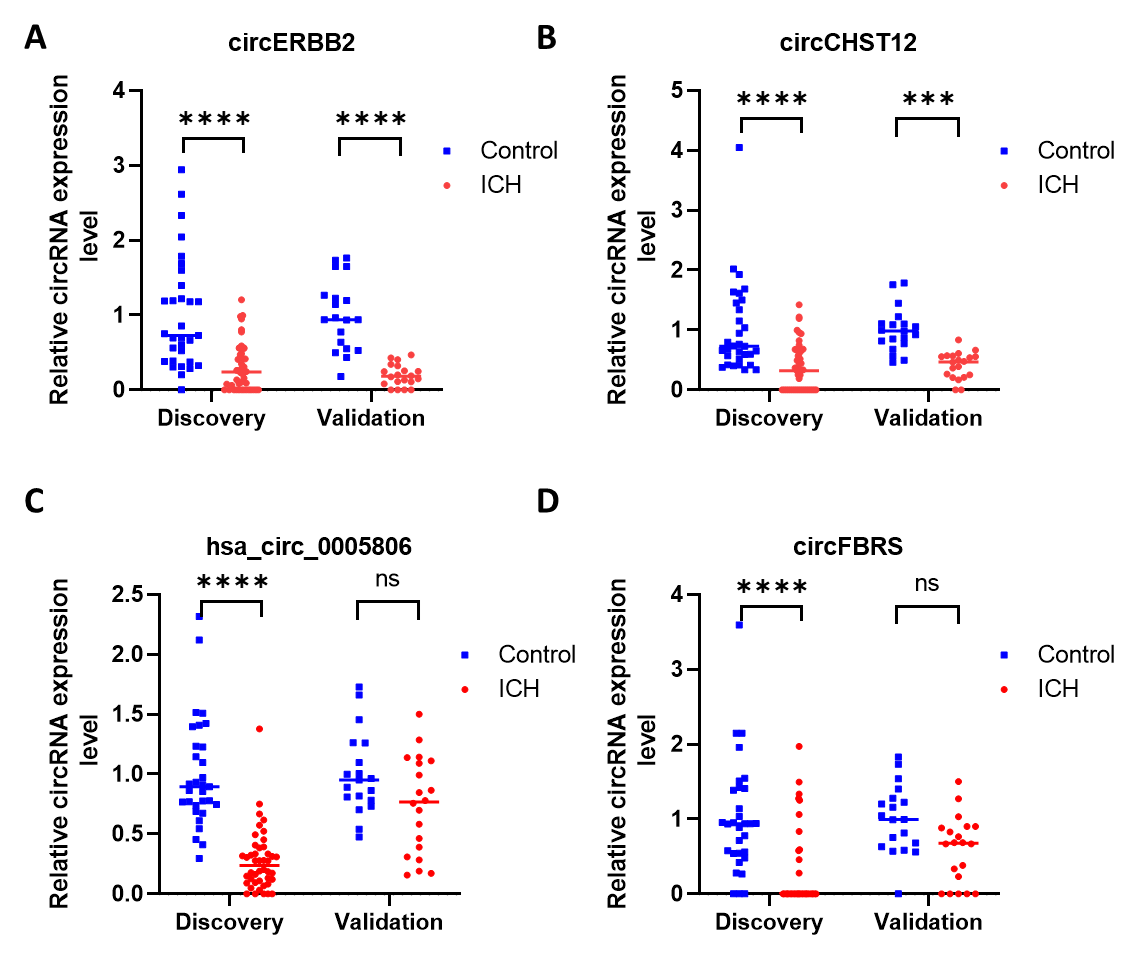
**

**Supplementary Figure 2. Relative expression levels of the four candidate circRNAs between intracerebral hemorrhage (ICH) patients and healthy controls in the discovery (n = 44 vs 31) and validation (n = 20 vs 19) cohorts.** (**A**) circERBB2, (**B**) circCHST12, (**C**) hsa_circ_0005806, (**D**) circFBRS. Data are presented as the median (interquartile range). *** p<0.001; **** p<0.0001; ns: no significance. Statistical significance was assessed using the Mann–Whitney U test.

1. **Supplementary Tables**

**Supplementary Tables 1-4 refer to the Supplemental Excel file.**

**Supplementary Table 1. Differentially Expressed circRNAs in ICH compared to healthy controls in the discovery cohort by the DESeq2 method.**

**Supplementary Table 2. Differentially Expressed circRNAs in ICH compared to healthy controls in the validation cohort by the DESeq2 method.**

**Supplementary Table 3. Differentially Expressed circRNAs in ICH compared to healthy controls in the discovery cohort by the EdgeR method.**

**Supplementary Table 4. Differentially Expressed circRNAs in ICH compared to healthy controls in the validation cohort by the EdgeR method.**

**Supplementary Table 5. The top 10 circRNA signatures in ICH with mutual information and random forest algorithms**

| Mutual information | Location | Coefficient | circRNA ID |
| --- | --- | --- | --- |
|  | chr7:2477438-2483381:+ | 0.534083815 | circCHST12 |
|  | chr16:30675536-30677862:+ | 0.531962677 | hsa_circ_0005806 |
|  | chr17:37866065-37872192:+ | 0.52143885 | circERBB2 |
|  | chr20:50133323-50140649:- | 0.480453133 | hsa_circ_0060849 |
|  | chr7:48541722-48542148:+ | 0.468119905 | hsa_circ_0001707 |
|  | chr16:30675536-30678943:+ | 0.44639756 | circFBRS |
|  | chrX:51638149-51640367:+ | 0.442615765 | hsa_circ_0008983 |
|  | chr12:66597491-66622150:+ | 0.44147719 | hsa_circ_0005505 |
|  | chr2:40655613-40657444:- | 0.438955066 | hsa_circ_0000994 |
|  | chr13:114817527-114822949:- | 0.429242065 | hsa_circ_0007514 |
| Random forest | Location | Coefficient | circRNA ID |
|  | chr16:30675536-30677862:+ | 0.011012 | hsa_circ_0005806 |
|  | chr2:74273405-74275538:+ | 0.005947 | hsa_circ_0006251 |
|  | chr16:30675536-30678943:+ | 0.005578 | circFBRS |
|  | chr17:37866065-37872192:+ | 0.0047 | circERBB2 |
|  | chr6:34574332-34614575:- | 0.0044 | hsa_circ_0009095 |
|  | chr16:4029117-4033441:- | 0.004365 | hsa_circ_0007586 |
|  | chr7:2477438-2483381:+ | 0.004072 | circCHST12 |
|  | chr7:155499554-155538296:+ | 0.003695 | circRBM33 |
|  | chr1:24993306-24996806:+ | 0.003417 | circSRRM1 |
|  | chr9:134070620-134077104:+ | 0.003401 | circNUP214 |

**Supplementary Table 6. Classification performance for the two-circRNA signatures between ICH and IS patients**

|  |  | Sensitivity (%) | | Specificity  (%) | Accuracy  (%) | PPV  (%) | NPV  (%) | AUC |
| --- | --- | --- | --- | --- | --- | --- | --- | --- |
| RF | Training set | 100.00 | | 98.34 | 99.21 | 98.50 | 100.00 | 0.989(0.971-1) |
|  | Test set | 64.88 | | 60.34 | 61.03 | 64.25 | 61.67 | 0.704(0.614-0.794) |
| KNN | Training set | 78.19 | 73.95 | | 76.28 | 76.94 | 75.75 | 0.832(0.761-0.903) |
|  | Test set | 57.52 | 52.98 | | 53.91 | 56.71 | 53.64 | 0.591(0.492-0.690) |
| DT | Training set | 100.00 | 98.34 | | 99.21 | 98.50 | 100.00 | 0.994(0.981-1) |
|  | Test set | 59.60 | 57.15 | | 56.35 | 58.08 | 60.01 | 0.549(0.448-0.650) |
| LR | Training set | 75.20 | 61.64 | | 68.78 | 68.33 | 69.37 | 0.761(0.678-0.844) |
|  | Test set | 74.50 | 60.01 | | 64.94 | 65.29 | 68.31 | 0.779(0.699-0.859) |
| GNB | Training set | 83.18 | 53.68 | | 69.13 | 66.40 | 74.29 | 0.761(0.678-0.844) |
|  | Test set | 83.43 | 53.25 | | 67.37 | 66.23 | 72.52 | 0.777(0.696-0.858) |
| SVM | Training set | 78.24 | 60.18 | | 69.67 | 68.36 | 71.60 | 0.762(0.679-0.845) |
|  | Test set | 76.73 | 61.12 | | 67.37 | 68.51 | 68.81 | 0.779(0.699-0.859) |

ICH: intracerebral hemorrhage; IS: ischemic stroke; RF: random forest; KNN: K-nearest neighbor; LR: logistic regression; DT: decision tree; GNB: Gaussian naive Bayes; SVM: support vector machine; PPV: positive predictive value; NPV: negative predictive value; AUC: area under the curve.

**Supplementary Table 7. Sequences of real-time PCR primers**

| **Primer Name** | **Sequence (5’-3’)** |
| --- | --- |
| hsa_circ_0001707-F | ATTGAGCTCCCCACAGAAAC |
| hsa_circ_0001707-R | TAGTAATTCCCCAACGTGCCAG |
| hsa_circ_0005505-F | AAGCCATTCACTACCTGCACA |
| hsa_circ_0005505-R | TATGACGAACATCCAGCCAGC |
| hsa_circ_0027725-F | TGAGTCAGGTGGCTTCACCC |
| hsa_circ_0027725-R | ACGGTGCCATAAACGGATGT |
| hsa_circ_0000914-F | CTGAAGCTCGACCACTACCG |
| hsa_circ_0000914-R | GTATGGGCTCCTGCTTGCC |
| circERBB2-F | CCCTCATCCACCATAACACCC |
| circERBB2-R | AGCGGGAGCCCTTACACATC |
| circCHST12-F | GCCGAGTTTGCCTCCTTGAA |
| circCHST12-R | TTAGCCACCCTGCGTTCAC |
| ERBB2-F | TGCAGGGAAACCTGGAACTC |
| ERBB2-R | ACAGGGGTGGTATTGTTCAGC |
| CHST12-F | GAGGTGAGGGTCGCGAGGTTC |
| CHST12-R | CACCTTGGGCACGTAGCAGT |
| GAPDH-F | GGTGAAGGTCGGAGTCAACG |
| GAPDH-R | CAAAGTTGTCATGGATGACC |
